# PRICKLE1, a Wnt/PCP signaling component, is overexpressed and associated with inferior prognosis in acute myeloid leukemia

**DOI:** 10.1186/s12967-021-02873-8

**Published:** 2021-05-17

**Authors:** Duanfeng Jiang, Yanjuan He, Qiuyu Mo, Enyi Liu, Xin Li, Lihua Huang, Qin Zhang, Fangping Chen, Yan Li, Haigang Shao

**Affiliations:** 1 Department of Hematology, The 3rd Xiangya Hospital, Central South University, Changsha, Hunan China; 2 Department of Hematology, Xiangya Hospital, Central South University, Changsha, Hunan China; 3 Department of Hematology, Affiliated Hospital of Guilin Medical University, Guilin, Guangxi China; 4 Center for Medical Experiments, The 3rd Xiangya Hospital, Central South University, Changsha, Hunan China

**Keywords:** Acute myeloid leukemia, Wnt/PCP signaling, PRICKLE1, Prognosis

## Abstract

**Background:**

Prickle planar cell polarity protein 1 (PRICKLE1), a core component of the non-canonical Wnt/planar cell polarity (PCP) pathway, was recently reported to be upregulated and correlated with poor prognosis in solid cancers. However, the effect of PRICKLE1 on acute myeloid leukemia (AML) remains unknown. This study aims to characterize the prognostic significance of PRICKLE1 expression in patients with AML.

**Methods:**

RNA-seq was performed to compare mRNA expression profiles of AML patients and healthy controls. qRT-PCR and western blotting were used to analyze the expression of PRICKLE1 in AML patients and cell lines, and two independent datasets (TCGA-LAML and TARGET-AML) online were used to validate the expression results. The correlations between the expression of PRICKLE1 and clinical features were further analyzed.

**Results:**

Our data showed that PRICKLE1 expression levels were markedly high in AML patients at the time of diagnosis, decreased after complete remission and increased again at relapse. Of note, PRICKLE1 was highly expressed in drug resistant AML cells and monocytic-AML patients. High PRICKLE1 expression was found in FLT3/DNMT3A/IDH1/IDH2-mutant AML and associated with poor prognosis. Furthermore, high expression of PRICKLE1 may be correlated with migration and invasion components upregulation in AML patients.

**Conclusions:**

These results indicated that high PRICKLE1 expression may be a poor prognostic biomarker and therapeutic target of AML.

**Supplementary Information:**

The online version contains supplementary material available at 10.1186/s12967-021-02873-8.

## Background

Acute myeloid leukaemia (AML) is a malignant disorder of haemopoietic stem cells characterized by clonal expansion of abnormally differentiated blasts of myeloid lineage [1]. In the past few years, benefited from translational research into genomic landscape, the therapeutic armamentarium of AML has expanded rapidly [2]. However, for most patients, primary and secondary drug resistance is still an urgent problem. In addition to improving our treatment strategies, our understanding of the biology and genomic structure of AML is deepening, which makes the risk assessment of AML more accurate and helps to choose appropriate treatment. For example, the favorable-risk mutation NPM1 and the adverse-risk mutation FLT3-ITD status interact to affect prognosis, and knowledge of both of these genes are required to fully assess relapse risk in an individual patient [1]. This emphasizes the necessity to identify molecular for providing new prognostic biomarkers and/or therapeutic targets.

Prickle planar cell polarity protein 1 (PRICKLE1) is a member of the non-canonical Wnt/planar cell polarity (PCP) pathway [2–4]. The Wnt/β-catenin pathway is required for the development of leukemia stem cells in AML [5–7], and its deregulation is involved in leukemia development [8]. Wnt/PCP signaling controls tissue polarity and cell movement and mediates collective migratory events [4, 9]. Components of Wnt/PCP signaling are often aberrantly expressed in solid cancers, and leads to abnormal activation of cancer cell migration pathways [3, 10, 11]. PRICKLE1 was recently described to be a poor-prognosis biomarker in breast cancer and be involved in metastatic dissemination [3, 12]. However, the role of PRICKLE1 remains largely unknown in AML.

In this study, the expression levels of PRICKLE1 were assessed in AML patients and cell lines by RNA-sequencing (RNA-seq), qRT-PCR and western blotting. Clinical prognostic significances were further investigated in AML patients with differential PRICKLE1 expression. To validate our findings, we performed the mRNA expression of PRICKLE1 using Gene Expression Profiling Interactive Analysis (GEPIA) online database, an AML cohort of 173 patients from the Cancer Genome Atlas-acute myeloid leukemia (TCGA-LAML) data and a non-M3 AML cohort of 145 patients from the the therapeutically applicable research to generate effective treatments (TARGET)-AML data. To understand the role of PRICKLE1 in AML, we explored the potential biological function of PRICKLE1 using a PRICKLE1-centered gene network and a protein–protein interaction network, which were analyzed by Search Tool for the Retrieval of Interacting Genes (STRING) and GeneMANIA databases.

## Methods

### Patients and clinical characteristics

Bone marrow (BM) samples and clinical data were obtained from patients who were diagnosed with AML between February 2017 and December 2019. The diagnosis and classifications of the patients were based on the French-American-British (FAB) classification [13] and 2016 WHO criteria [14]. Samples were collected from patients at different stages of AML, including patients with newly diagnosed AML (n = 129), relapsed AML (n = 13) and complete remission (CR) (n = 35). Relapsed and CR were defined according to the European LeukemiaNet (ELN) recommendations [15]. Control samples (n = 12) were obtained from donors without any malignant BM disorder, containing 3 haploidentical healthy donors. BM mononuclear cells were isolated using Ficoll-Hypaque (Sigma-Aldrich, St Louis, MO) density gradient separation. Informed consent was obtained according to the Declaration of Helsinki. The use of BM samples was approved by the Medical Ethics Committee of the Third Xiangya Hospital of Central South University.

### Cell lines and cell culture

The human myeloid leukemia cell lines K562, K562/ADR, THP1, HL60, HL60/ADR and human normal hematopoietic cell line GM12878 were obtained from the Cancer Research Institute of Central South University. The human lymphocytic leukemia cell line Jurkat was purchased from Cell Bank of Chinese Academy of Sciences (Shanghai, China). The MOLM13 and MV4-11 cell lines, were provided by Professor Hui Zeng working in the First Affiliated Hospital of Jinan University (Guangzhou, China). All cells were grown in RPMI 1640 (Gibco, USA) medium, supplemented with 10% fetal bovine serum (Gibco, USA) at 37 ºC in a 5% CO_2_ incubator. HL60/ADR and K562/ADR cells were cultured in the presence of adriamycin (1 μmol/L).

### Separation and enrichment of CD34^+^ cells

Samples used for RNA-seq need CD34^+^ sorting. BM mononuclear cells from healthy donors and AML patients were isolated by Ficoll-Hypaque (Sigma-Aldrich) density gradient separation. And then CD34^+^ cells were enriched using a Miltenyi microbead separation system (Miltenyi BioTech, Auburn, CA) according to the manufacturer's instructions. The purity of the isolated CD34^+^ cells was determined by flow cytometry (Becton Dickinson, USA).

### Western blotting analysis

Total proteins were extracted using RIPA buffer (NCM Biotech) with freshly added proteinase inhibitor. Proteins were then separated by 10–12% SDS/PAGE and transferred to 0.22 μm PVDF membranes (Millipore). The membranes were blocked with 5% skim milk and then incubated with primary antibodies overnight at 4 ºC. The primary antibodies used in this study were anti-PRICKLE1 (Proteintech, USA, 22589–1-AP) used at 1:1000, anti-DVL2 (Affinity, USA, DF4454) used at 1:1000, anti-LEF1 (Affinity, USA, DF7570) used at 1:1000, anti-Active β-catenin (Sigma-Aldrich, USA, 05-665) used at 1:1000, or anti-β-actin (Affinity, USA, DF7018) used at 1:1500. Appropriate HRP-conjugated secondary antibodies, and protein signals were developed with the enhanced chemiluminescence (ECL) reagents (Affinity Biosciences). ChemiDox XRS Chemiluminescence imaging system (Bio-Rad, USA) was used to capture and analyze the developed images.

### Quantitative reverse transcription-polymerase chain reaction (qRT-PCR)

Total RNA was isolated using Trizol reagent (Invitrogen, USA) and converted to cDNA using HiScript III RT SuperMix for qPCR (Vazyme, Nanjing, China). Gene-specific primers were synthesized by the Beijing Genomics Institute. Gene expression (mRNA) was analysed using the ChamQ Universal SYBR qPCR Master Mix (Vazyme, #Q711) and LightCycler 480 real-time PCR instrument (Roche, Switzerland) in a two-step qRT-PCR (95 ºC for 30 s, followed by 40 cycles of 95 ºC for 10 s and 60 ºC for 30 s). The mRNA relative levels of the target genes were calculated using the 2^−ΔΔCt^ method, clinical samples using ABL1 and cell lines using β-actin as the endogenous control. The data were obtained by normalizing PRICKLE1 gene Ct values with reference gene Ct values, and then analyzed with 2^−ΔΔCt^ method. Primers used in this study were as follows: PRICKLE1, forward 5′-TGCTGCCTTGAGTGTGAAAC-3′, reverse 5′-CACAAGAAAAGCAGGCTTCC-3′; ABL1, forward 5′-GATACGAAGGGAGGGTGTACCA-3′, reverse 5′-CTCGGCCAGGGTGTTGAA-3′; β-actin, forward 5′-GGACTTCGAGCAAGAGATGG -3′, reverse 5′-AGCACTGTGTTGGCGTACAG-3′.

### RNA-seq

Control samples (n = 3) for RNA-seq were obtained from haploidentical healthy donors before mobilization of hemopoietic stem cells. RNA sample quality was analysed, and the cDNA libraries were synthesized and sequenced using BGI technology [16]. Briefly, the quality of the RNA samples was assessed by an Agilent Bioanalyzer (Agilent). cDNA libraries were generated using TruSeq RNA Sample Preparation (Illumina). Each library was sequenced using single-reads on a HiSeq2000/1000 (Illumina). Gene expression levels were measured in RPKM using Cufflinks [17]. Differentially expressed genes (DEGs) were identified using the DESeq2 R package. The criteria for DEGs was set up as fold change (FC, log2) > 2 or <  − 2, Q-value < 0.05, and FDR < 0.05. RNA sequencing data were analysed by Partek Inc. (St. Louis, MO).

### Online source

GEPIA Dataset, the expression differences of PRICKLE1 between AML patients and normal controls were conducted by GEPIA dataset (http://gepia.cancer-pku.cn/detail.php) [18, 19]. Two independent datasets (Cohort 1: TCGA-LAML; Cohort 2: TARGET-AML) were obtained from The Cancer Genome Atlas (TCGA) (https://cancergenome.nih.gov/ and http://www.cbioportal.org/) [20]. Cohort 1 consisted of samples from 173 adult AML patients (including 157 non-M3 AML) and Cohort 2 comprised of 145 primary non-M3 AML patients. The RNA-seq data and survival data were extracted for further analysis. The online website of GeneMANIA (http://genemania.org/) [21] was used to construct the PRICKLE1 centered gene–gene functional interaction network. Functional and signaling pathway enrichment was conducted using online site of STRING (http://string-db.org) [19].

### Statistical analyses

The differences between continuous variables were using unpaired t test or the Mann–Whitney U test. Comparisons in proportions of variables between two groups were analyzed using the χ^2^ test. Paired Wilcoxon was used to analyze the difference between before- and after- treatment. In the TARGET-AML database, event-free survival (EFS) was measured from diagnosis until the patient experienced induction failure, relapse or death. In the TCGA-AML database and our cohort, EFS was defined as the day from diagnosis to relapse or death. In all cohort, overall survival (OS) was defined as the day from diagnosis to last follow-up or death [22]. EFS and OS was analyzed though Kaplan–Meier analysis using Log-rank test. Univariate and multivariate analyses were performed using the Cox proportional hazard model. For all tests, a P-value < 0.05 indicated statistical significance. Statistical analysis was performed using SPSS 19.0, R software 3.5.0. and GraphPad Prism 7.0.

## Results

### Transcriptional levels of PRICKLE1 in normal controls, AML patients and cell lines

**Fig. 1:**
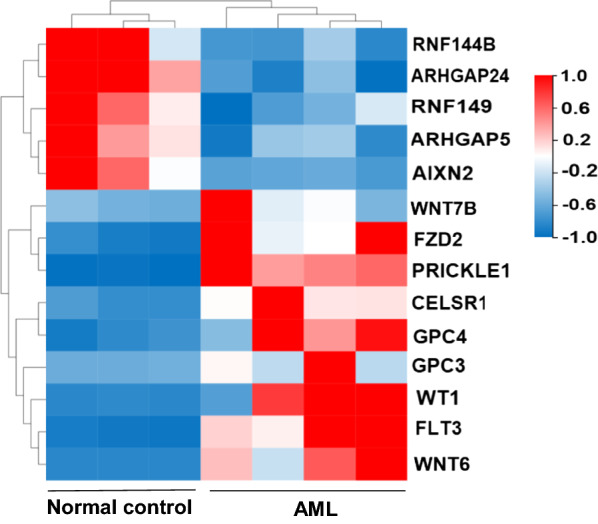
Gene expression detected by RNA-Seq and expression of PRICKLE1 in AML patients. Hierarchical cluster analysis of DEGs in AML patients (n = 4) and normal controls (n = 3), wnt signaling pathway related genes were shown. Upregulated genes are shown in red and downregulated genes are shown in blue

**Fig. 2:**
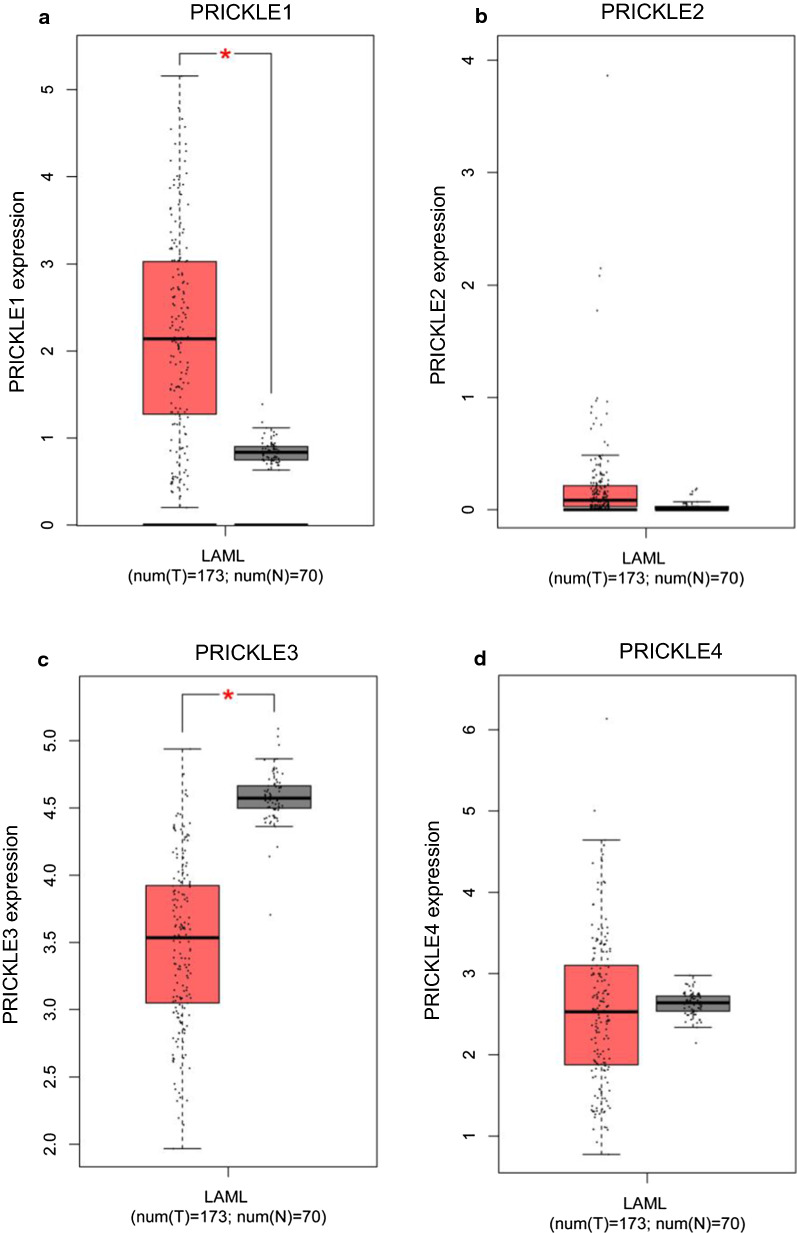
Expression differences of PRICKLE1 (**a**), PRICKLE2 (**b**), PRICKLE3 (**c**), and PRICKLE4 (**d**) between 173 de novo AML patients and 70 normal controls. Analysis with the GEPIA computer tool and the data from the TCGA database. *P < 0.01

**Fig. 3:**
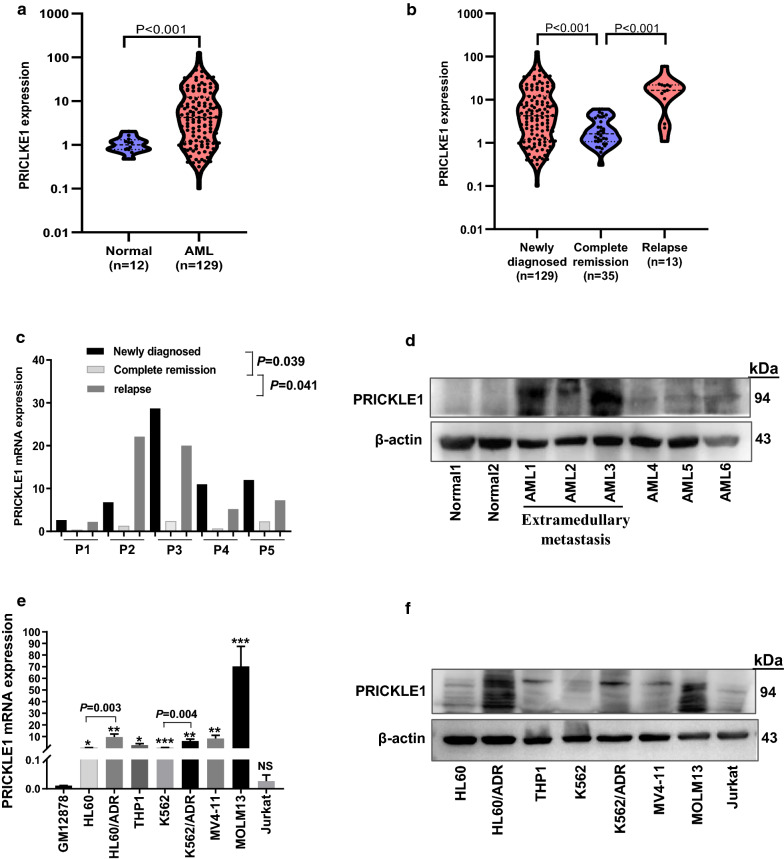
Expression of PRICKLE1 in primary AML samples and cell lines. **a** qRT-PCR analysis of PRICKLE1 mRNA expression in AML patient samples (n = 129) and normal controls (n = 12). **b** PRICKLE1 mRNA expression in patients with newly-diagnosed AML (n = 129), patients with relapsed AML (n = 13) and patients with complete remission (n = 35). **c** qRT-PCR analysis of 5 patients with AML at the time of newly diagnosed, complete remission and relapse. **d** Representative western blotting analysis of PRICKLE1 in patients with AML (n = 6) including patients with extramedullary metastasis (n = 3) relative to control (n = 2). **e**, **f** qRT-PCR and western blotting analysis of PRICKLE1 expression in AML cell lines. GM12878 cell line was used as the control group. *P < 0.05; **P < 0.01; ***P < 0.001; NS: not significant

We first screened the differentially expressed genes (DEGs) of Wnt pathway between four AML patients and three healthy donors using RNA-seq analysis. The results indicated the expression of PRICKLE1 was markedly elevated in AML patients compared with normal controls (Fig. 1 and Additional file 1: Table S1). These findings are supported by the results of analysis with the GEPIA computer tool and the data from the TCGA database (Fig. 2a–d). Subsequently, PRICKLE1 mRNA levels were examined in bone marrow (BM) samples from AML patients (n = 129) and samples from normal controls (n = 12). Clinical characteristics were summarized in Tables 1 and 2. Compared to normal controls, the mRNA of PRICKLE1 was significantly upregulated in AML (P < 0.001, Fig. 3a). In addition, higher PRICKLE1 expression was observed in patients with newly diagnosed AML (n = 129, P < 0.001) and relapsed AML (n = 13, P < 0.001) than in patients with complete remission (n = 35, Fig. 3b). Furthermore, PRICKLE1 protein was generally expressed in AML patients (Additional file 1: Table S2) compared to normal controls, especially was highly expressed in the patients with extramedullary metastasis (Fig. 3d and Additional file 1: Fig. S1). It is worth noting that, PRICKLE1 mRNA levels were determined in 5 patients at the time of being newly diagnosed, complete remission and relapse. A common feature was that the expression of PRICKLE1 was high at new diagnosis, decreased after complete remission (P = 0.039, Fig. 3c), and increased again at relapse (P = 0.041, Fig. 3c).

**Table 1:** Correlation of PRICKLE1 expression with clinical and laboratorial parameters in AML patients

Patient's parameters	Total (n = 129)	Status of PRICKLE1 expression		P value
		High (n = 65)	Low (n = 64)	
Sex, male/female	77/52	35/30	42/22	0.236
Median age, years (range)	51 (12–81)	52 (14–81)	50(12–80)	0.421
Median WBC, × 10^9^/L (range)	15.60(0.30–381.60)	19.90 (0.30–381.60)	13.31 (0.65–263.45)	0.260
Median hemoglobin, g/L (range)	73 (33–151)	73 (33–141)	75 (36–151)	0.642
Median platelets, × 10^9^/L (range)	30 (2–984)	32 (2–277)	27 (5–984)	0.456
Median BM blasts %, (range)	73.0 (16.0–97.0)	78.0 (16.0–97.0)	66.0 (21.0–97.0)	0.036
FAB subtypes (%)				< 0.001
M0	1 (0.8)	0 (0.0)	1 (1.6)	
M1	13 (10.1)	6 (9.2)	7 (10.9)	
M2	49 (38.0)	13 (20.0)	36 (56.3)	
M3	17 (13.2)	12 (18.5)	5 (7.8)	
M4	13 (10.1)	6 (9.2)	7 (10.9)	
M5	34 (26.4)	27 (41.6)	7 (10.9)	
Not determined	2 (1.6)	1 (1.5)	1 (1.6)	
ELN risk stratification (%)				0.095
Favorable	50 (38.8)	21 (32.3)	29 (45.3)	
Intermediate	44 (34.1)	20 (30.8)	24 (37.5)	
Adverse	19 (14.7)	13 (20.0)	6 (9.4)	
No data	16 (12.4)	11 (16.9)	5 (7.8)	
Cytogenetic risk (%)				0.304
Favorable	39 (30.2)	17 (26.2)	22 (34.4)	
Intermediate	59 (45.7)	28 (43.1)	31 (48.4)	
Adverse	15 (11.6)	9 (13.8)	6 (9.4)	
No data	16 (12.4)	11 (16.9)	5 (7.8)	
Karyotypes (%)				0.023
t(8;21)/RUNX1-RUNX1T1	18 (14.0)	4 (6.2)	14 (21.9)	
inv(16)/CBFβ-MYH11	4 (3.1)	1 (1.5)	3 (4.7)	
t(15;17)/PML-RARA	17 (13.2)	12 (18.5)	5 (7.8)	
11q23/MLL	7 (5.4)	6 (9.2)	1 (1.6)	
Normal karyotype	41 (31.8)	19 (29.2)	22 (34.4)	
Complex karyotype	5 (3.9)	2 (3.1)	3 (4.7)	
Other karyotype	21 (16.3)	10 (15.4)	11 (17.2)	
No data	16 (12.4)	11 (16.9)	5 (7.8)	
FLT3 (%)				0.007
FLT3-ITD	16 (12.4)	14 (21.5)	2 (3.1)	
FLT3-TKD	4 (3.1)	2 (3.1)	2 (3.1)	
Wild	85 (65.9)	40 (61.6)	45 (70.3)	
No data	24 (18.6)	9 (13.8)	15 (23.4)	
CEBPA (%)				< 0.001
Single mutation	3 (2.3)	2 (3.1)	1 (1.6)	
Double mutation	15 (11.6)	1 (1.5)	14 (21.9)	
Wild	87 (67.4)	53 (81.6)	34 (53.1)	
No data	24 (18.6)	9 (13.8)	15 (23.4)	
DNMT3A (%)				0.023
Mutated	6 (4.7)	6 (9.2)	0 (0.0)	
Wild	99 (76.7)	50 (77.0)	49 (76.6)	
No data	24 (18.6)	9 (13.8)	15 (23.4)	
IDH1 (%)				0.079
Mutated	7 (5.4)	6 (9.2)	1 (1.6)	
Wild	98 (76.0)	50 (77.0)	48 (75.0)	
No data	24 (18.6)	9 (13.8)	15 (23.4)	
IDH2 (%)				0.002
Mutated	10 (7.8)	10 (15.4)	0 (0.0)	
Wild	95 (73.6)	46 (70.8)	49 (76.6)	
No data	24 (18.6)	9 (13.8)	15 (23.4)	
NPM1 (%)				< 0.001
Mutated	23 (17.8)	20 (30.8)	3 (4.7)	
Wild	82 (63.6)	36 (55.4)	46 (71.9)	
No data	24 (18.6)	9 (13.8)	15 (23.4)

**Table 2:** Patients’ information

Patient’s characteristics	Normal control (n = 12)	AML-CR (n = 35)	AML-Relapse (n = 13)
Median age in years (range)	29 (20–42)	42 (18–80)	54 (20–68)
Sex (male/female)	5 (41.7%)/7 (58.3%)	21 (60%)/14 (40%)	4 (30.8%)/9 (69.2%)
Unfavorable fusion gene	–	3 (8.6%)	5 (38.5%)
Unfavorable karyotype	–	3 (8.6%)	4 (30.8%)

Next, we explored the expression of PRICKLE1 in leukemia cell lines using qRT-PCR and western blotting. Compared with the control cell line GM12878 (Fig. 3e), the expression of PRICKLE1 was significantly upregulated in 7 AML cell lines (P < 0.05), but not in human T cell acute lymphoblastic leukemia cell line, Jurkat cells (P > 0.05). Among AML cell lines, K562 and HL60 showed lower mRNA (Fig. 3e) and protein (Fig. 3f) levels of PRICKLE1 than their corresponding adriamycin-resistant cell lines, K562/ADR and HL60/ADR (K562 vs K562/ADR, P = 0.004; HL60 vs HL60/ADR, P = 0.003). Therefore, PRICKLE1 may play an important role in AML drug resistance.

### High PRICKLE1 expression is correlated with BM blasts, FAB classifications and poorer risk classification in AML patients

To explore the correlation of PRICKLE1 expression with clinical features in AML patients, we divided the patients into a high PRICKLE1 expression group (PRICKLE1^high^, the first half, n = 65) and a low PRICKLE1 expression group (PRICKLE1^low^, the second half, n = 64) according to the cut-off value of 4.25 (median PRICKLE1 expression level). The comparisons of clinical features and laboratory parameters between the two groups were shown in Table 1. The high expression of PRICKLE1 was found to be associated with higher BM blasts (P = 0.036). Remarkable differences were also observed in the distributions of FAB classifications (P < 0.001) and karyotypes (P = 0.023). Moreover, the expression level of PRICKLE1 in monocytic-AML was higher than that in granulocytic-AML (Additional file 1: Fig. S2a). However, we did not observe significant differences in sex, age, white blood cells (WBCs), hemoglobin (HB), platelets (PLT), and cytogenetic risk between PRICKLE1^high^ and PRICKLE1^low^ patients (Table 1).

**Fig. 4:**
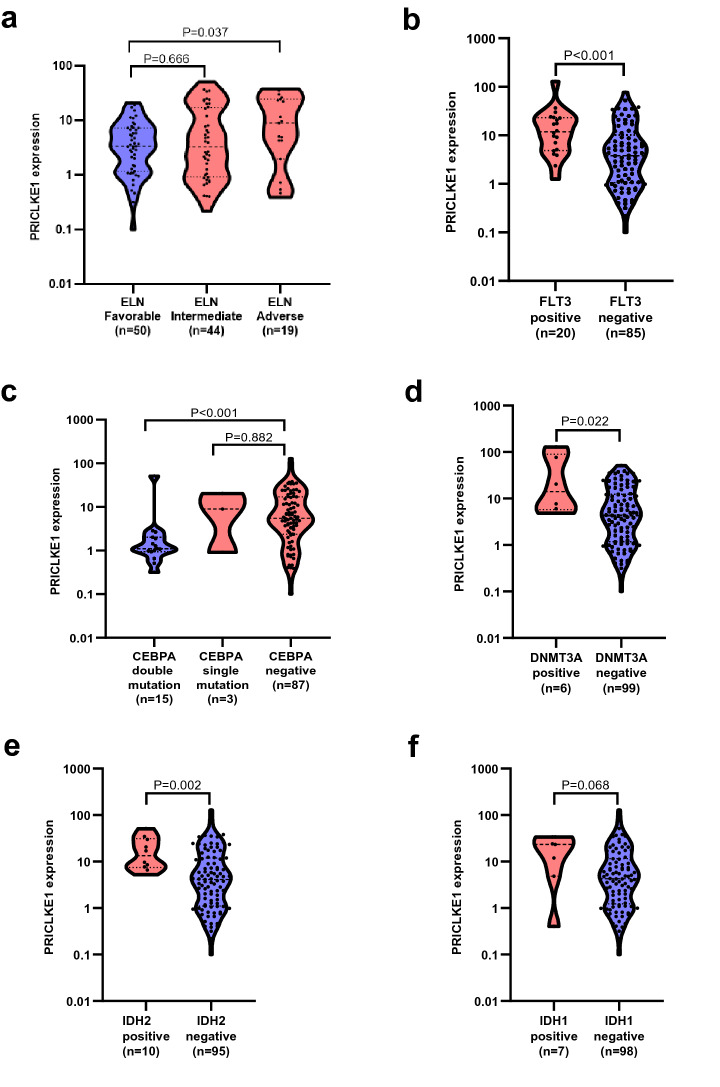
Association of PRICKLE1 expression with European LeukemiaNet (ELN) risk classification in AML patients. PRICKLE1 expression difference between patients with good-risk cytogenetic classification and intermediate/poor-risk cytogenetic classification (**a**), and patients with gene mutations [FLT3 (**b**), CEBPA (**c**), DNMT3A (d), IDH2 (**e**) and IDH1 (**f**)]

Interestingly, we found that patients with poor European LeukemiaNet (ELN) risk [1, 15] had much higher PRICKLE1 expression compared with patients with good risk (P = 0.037, Fig. 4a). Furthermore, high expression of PRICKLE1 was found to be associated with unfavorable gene mutations FLT3 (P < 0.001, Fig. 4b), DNMT3A (P = 0.022, Fig. 4d) and IDH2 (P = 0.002, Fig. 4e), and tended to be associated with IDH1 (P = 0.068, Fig. 4f). Consistently, low expression of PRICKLE1 was related to favorable gene mutation CEBPA double mutation (P < 0.001, Fig. 4c), but not to CEBPA single mutation (P = 0.882, Fig. 4c). It is noteworthy that, patients with NPM1 mutation also showed high expression of PRICKLE1 (P < 0.001, Additional file 1:Fig. S2b), which is often concomitant with FLT3, IDH1/2, and DNMT3A mutations [1].

### High PRICKLE1 expression is an independent prognostic indicator of adverse outcomes in patients with AML

**Fig. 5:**
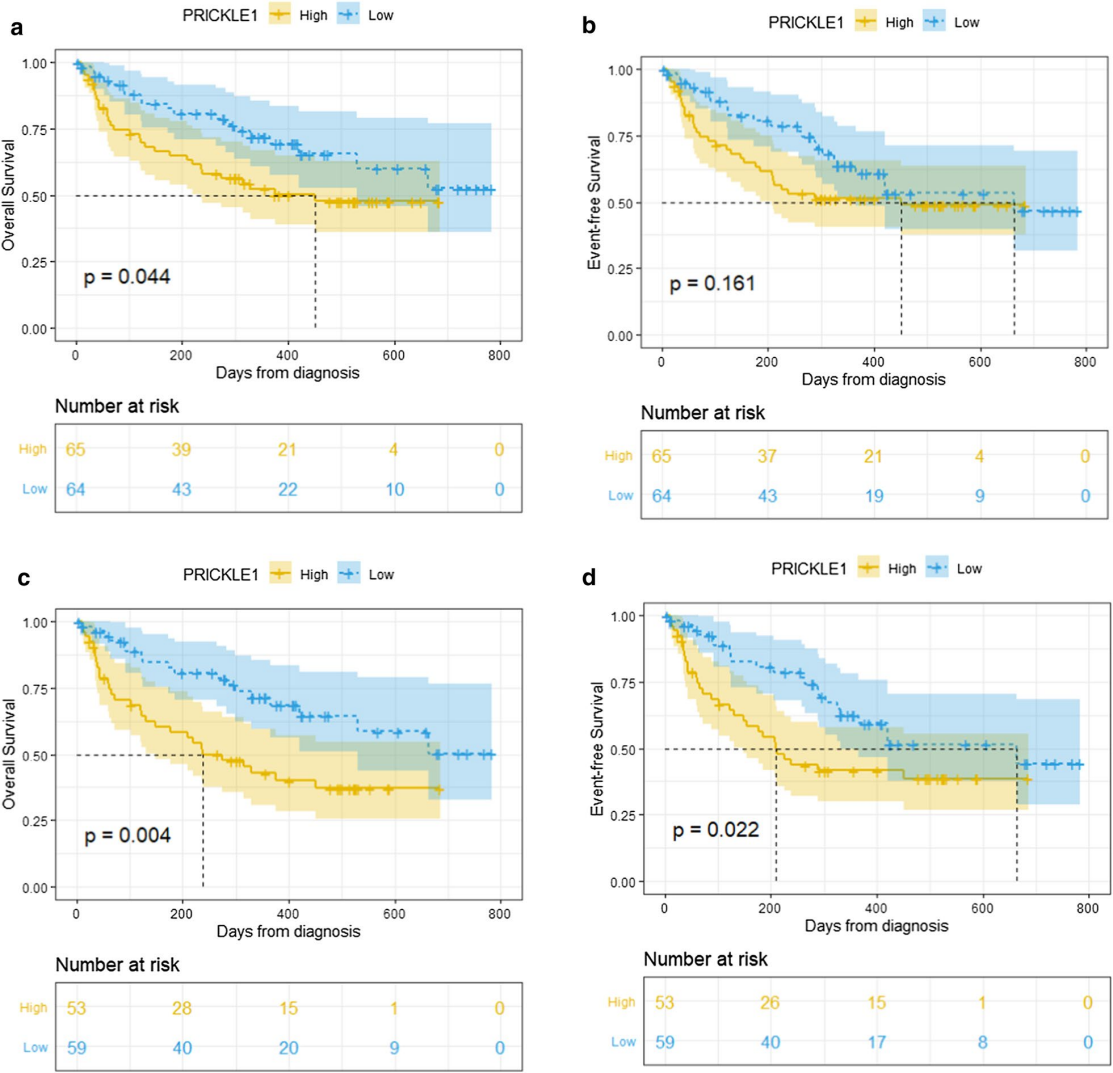
Survival analysis of AML patients in our study according to PRICKEL1 expression. **a** Overall survival (OS) of whole-cohort AML patients; **b** Event-free survival (EFS) of whole-cohort AML patients; **c** OS of non-M3 AML patients; **d** EFS of non-M3 AML patients

In this study, 129 patients who can be evaluated were received median follow-up period of 10 months (1–26 months). Kaplan–Meier survival analysis showed that patients with high PRICKLE1 expression (n = 65) had significantly shorter overall survival (OS) (P = 0.044, Fig. 5a) than those of patients with low PRICKLE1 expression (n = 64) in the whole-cohort AML patients. Among the 112 non-M3 AML patients, PRICKLE1^high^ cases (n = 53) also showed significantly shorter OS and event-free survival (EFS) than PRICKLE1^low^ cases (n = 59) (OS: P = 0.004, Fig. 5c; EFS: P = 0.022, Fig. 5d). Although there were no significant difference in EFS between PRICKLE1^high^ and PRICKLE1^low^ patients with whole-cohort AML (P = 0.161, Fig. 5b), and in OS between PRICKLE1^high^ and PRICKLE1^low^ patients with cytogenetically normal AML (CN-AML) (P = 0.300, Additional file 1: Fig. S3a), a trend of separation will emerge with the extension of follow-up time.

**Fig. 6:**
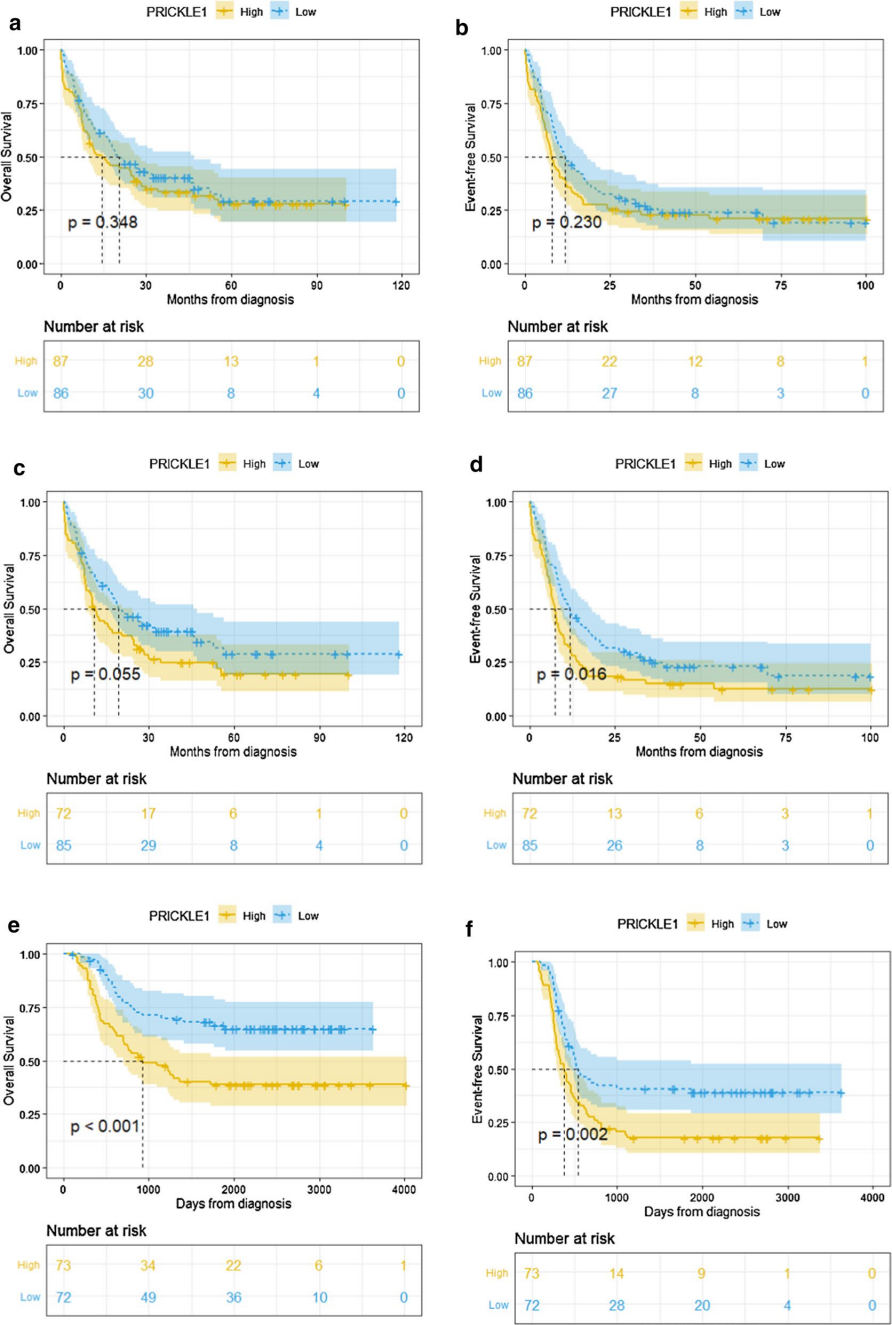
Survival analysis of AML cases from data online according to PRICKEL1 expression. **a** OS of whole-cohort AML in TCGA-LAML data; **b** EFS of whole-cohort AML in TCGA-LAML data; **c** OS of non-M3 AML in TCGA-LAML data; **d** EFS of non-M3 AML in TCGA-LAML data. **e** OS of non-M3 AML in TARGET-AML data; **f** EFS of non-M3 AML in TARGET-AML data

Further, we analyzed an AML cohort of 173 patients including 157 non-M3 AML patients from the TCGA-LAML data and a non-M3 AML cohort of 145 patients from the TARGET-AML data [20, 23]. We reached the same conclusion that PRICKLE1^high^ cases showed an markedly shorter OS (TCGA-LAML data: P = 0.055, Fig. 6c; TARGET-AML data: P < 0.001, Fig. 6e) and EFS (TCGA-LAML data: P = 0.016, Fig. 6d; TARGET-AML data: P = 0.002, Fig. 6f) compared with PRICKLE1^low^ cases in the non-M3 AML patients. However, between PRICKLE1^high^ and PRICKLE1^low^ patients from TCGA-LAML data, there were no significant difference in OS (P = 0.348, Fig. 6a) and EFS (P = 0.230, Fig. 6b) of whole-cohort AML or in OS of CN-AML (P = 0.490, Additional file 1: Fig. S3b).

We then performed univariate analyses and multivariate analyses on OS in the total 129 AML patients, including the expression level of PRICKLE1, age, WBC, cytogenetic risk and NPM1/FLT3-ITD/CEBPA/DNMT3A/IDH1/IDH2 mutations (mutant vs. wild-type). As shown in Table 3, PRICKLE1 expression was significantly and independently associated with a worse OS both in univariate (P = 0.005) and multivariate analysis (P = 0.012). Besides, age and cytogenetic risk were related to poorer OS both in univariate analysis (P = 0.006; P = 0.020; respectively).

**Table 3:** Results of univariate and multivariate analysis for OS in non-M3 AML patients

	Univariate		Multivariate	
	HR (95% CI)	P value	HR (95% CI)	P value
PRICKLE1 (high vs. low)	2.324 (1.289–4.192)	0.005	3.087 (1.288–7.397)	0.012
Age (> median vs. < median)	2.272 (1.270–4.067)	0.006	2.028 (0.896–4.590)	0.090
WBC (> median vs. < median)	1.508 (0.828–2.746)	0.179	1.743 (0.752–4.040)	0.195
Cytogenetic risk (poor vs. intermediate vs. favorable)	1.792 (1.095–2.935)	0.020	1.259 (0.739–2.144)	0.397
NPM1 (mutated vs. wild)	0.990 (0.451–2.174)	0.980	0.378 (0.099–1.445)	0.155
FLT3-ITD (mutated vs. wild)	1.379 (0.572–3.321)	0.474	0.923 (0.226–3.770)	0.912
CEBPA (mutated vs. wild)	0.400 (0.141–1.132)	0.084	0.407 (0.132–1.255)	0.118
DNMT3A (mutated vs. wild)	0.750 (0.180–3.128)	0.693	0.688 (0.082–5.793)	0.731
IDH1 (mutated vs. wild)	1.449 (0.509–4.123)	0.487	0.571 (0.159–2.047)	0.389
IDH2 (mutated vs. wild)	0.806 (0.246–2.641)	0.721	0.527 (0.106–2.618)	0.433

OS: Overall survival; HR: hazard ratio; CI: confidence interval; WBC: white blood cell

### High expression of PRICKEL1 accompanied with core PCP pathway components upregulation in AML patients

**Fig. 7:**
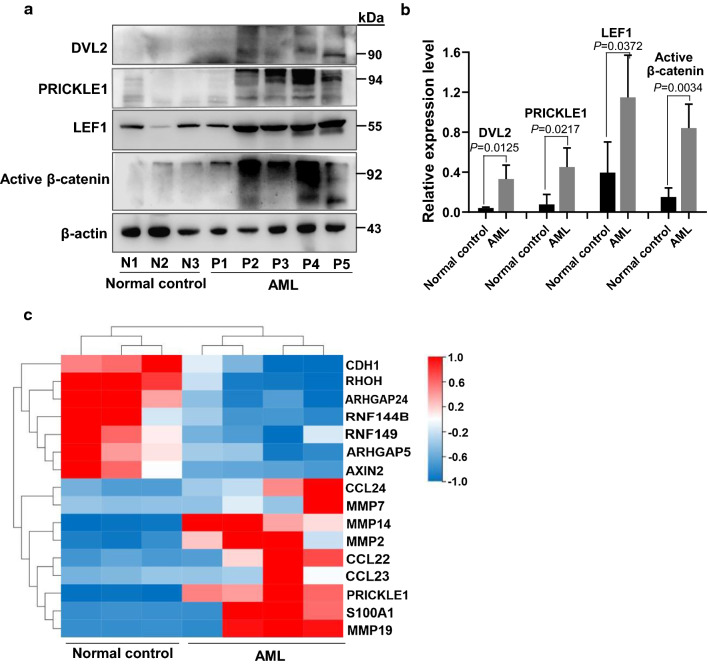
High expression of PRICKEL1 accompanied with core PCP pathway components and the migration and invasion components up-regulation in AML patients. **a** The amounts of the indicated PCP proteins were analyzed by Western blotting in AML patients (n = 5) and normal controls (n = 3). **b** Densitometric quantification of data in **a**, the expression of the PCP proteins DVL2, PRICKLE1, LEF1 and active β-catenin were analyzed. **c** RNA-Seq analysis of gene expression in AML patients. Hierarchical cluster analysis of DEGs in AML patients (n = 4) and normal controls (n = 3), migration and invasion related genes were shown

Human WNT5A, WNT5B, and WNT11 are representative non-canonical WNTs transducing PCP signals through FZD3 or FZD6 receptors, and ROR1, ROR2 or PTK7 co-receptors. Human VANGL1, VANGL2, CELSR1, CELSR2, CELSR3, DVL1, DVL2, DVL3 (Dishevelled homologs), PRICKLE1, PRICKLE2, and ANKRD6 are core PCP signaling components [9, 24]. As our above data have shown that PRICKEL1 is distinctly upregulated in AML patients, we further analyzed the expression of the other core PCP signaling components. RNA-seq analysis showed that WNT6, WNT7B, FZD2, PRICKLE1 and CELSR1 were significantly upregulated in AML patients; while WNT signaling inhibitors, such as AXIN2 [25], were downregulated (Fig. 1). Furthermore, the protein levels of DVL2, PRICKLE1, LEF1 and active β-catenin were increased in AML patients compared with normal control (Fig. 7a,b). These results revealed that PCP pathway may be activated in PRICKLE1^high^ AML patients.

### High expression of PRICKEL1 accompanied with the migration and invasion components upregulation in AML patients

The planar cell polarity (PCP) protein PRICKLE1, ArhGAP21/23 and the RhoGAPs are involved in coordinating shape volatility during productive cell migration [26]. Our above data showed that PRICKLE1 protein levels were higher than normal control (Fig. 3d). Further RNA-seq analysis indicated that a series of metastasis and invasion molecules were upregulated in PRICKLE1-high AML patients, such as CCL22, CCL23, CCL24, MMP2, MMP7, MMP14, MMP19 and S100A1; while invasion suppressors, such as CDH1 [27], were downregulated in PRICKLE1^high^ AML patients (Fig. 7c and Additional file 1: Table S3).

### Bioinformatic analysis of PRICKLE1 function in AML

**Fig. 8:**
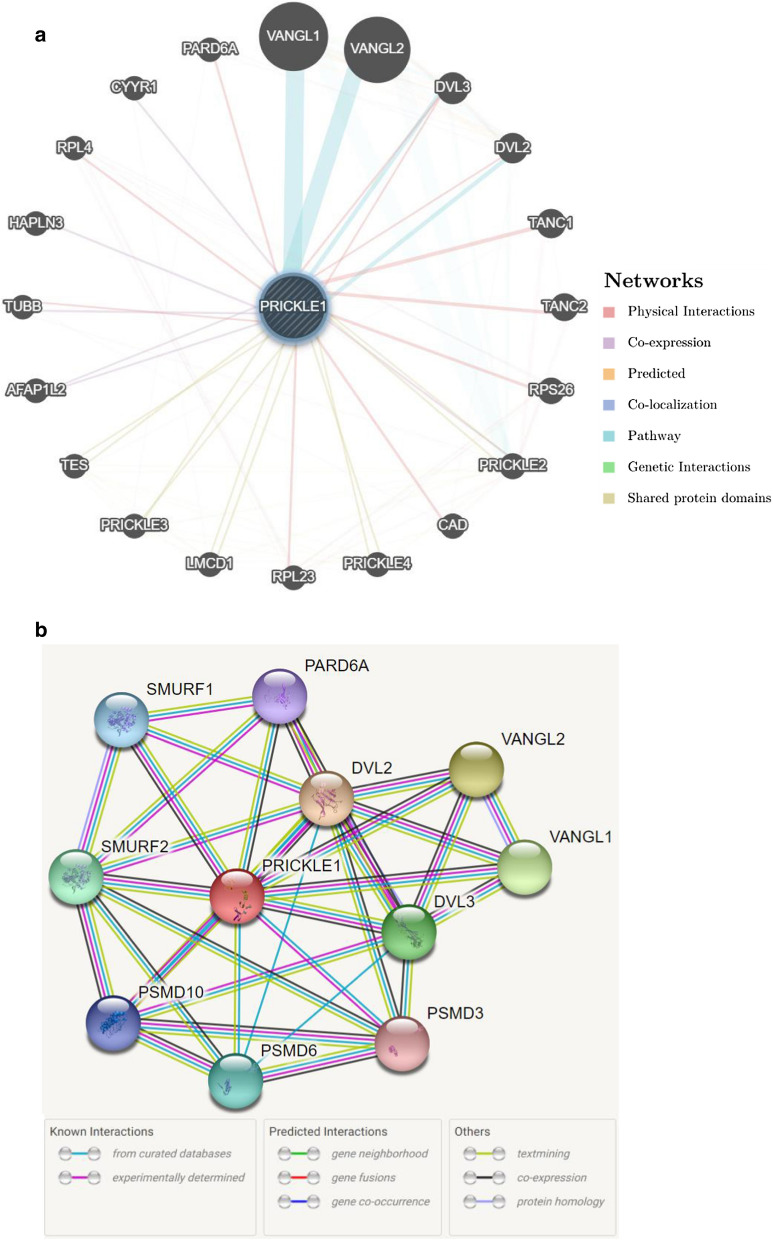
Potential biological functions of PRICKLE1. The potential regulatory network of PRICKLE1 in AML analzed by GeneMANIA and STRING. **a** PRICKLE1 centered gene–gene functional interaction network analyzed by GeneMANIA, showing the genes with physical interactions, shared signaling path ways, and predicted interactions with PRICKLE1. **b** Protein–protein interaction network of PRICKLE1 analyzed by STRING. Color images are available online

To investigate the functional roles of PRICKLE1, we constructed a PRICKLE1-centered network showing genes in AML using GeneMANIA (Fig. 8a). Results showed that VANGL1, VANGL2, DVL2 and DVL3 have shared signaling pathways with PRICKLE1; moreover, DVL2 and DVL3 also have physical interactions with PRICKLE1. PRICKLE2, PRICKLE3 and PRICKLE4 have predicted interactions with PPRICKLE1. The protein–protein interactions of PRICKLE1 with other partners in AML were analyzed using STRING online tools. The results showed that PRICKLE1 interacted with SMURF1 and SMURF2, which play a key roll in the regulation of cell motility, cell signalling, and cell polarity [26]. (Fig. 8b). In addition, PRICKLE1 interacted with VANGL1, VANGL2, DVL2 and DVL3, which are in line with the results of gene network analysis. Of note, PRICKLE1 interacted with PARD6A, a cell membrane protein which plays a role in cell polarization and the epithelial-to-mesenchymal transition (EMT) that represents the invasive phenotype in metastatic carcinomas [28]. In general, these data shows that PRICKLE1 may be involved in cell polarization and migration.

## Discussion

AML is a heterogeneous disease characterized by extensive molecular changes that affect clinical outcomes and provide potential targets for drug development [1, 2], such as targeting the FLT3 mutations in AML [29]. Genetic abnormalities are powerful prognostic factors [30, 31]. However, models incorporating genomic lesions, demographic, clinic and treatment and aimed at predicting are correct in only about 75% of cases [30]. This emphasizes the need to identify other prognostic factors. It is known that Wnt/β-catenin signaling pathway is required for self-renewal and function of leukemia stem cells (LSCs) in AML [5]. The nine upregulation genes and the four downregulation genes (Fig. 1) from our results of RNA-seq were selected for further investigation on the basis of their relevance to Wnt/β-catenin signaling pathway of AML.

In the present study, we found that PRICKLE1 expression was significantly increased in newly diagnosed or relapsed AML patients compared with normal controls, which was consistent with the results of RNA-seq. Moreover, GEPIA analysis indicated that PRICKLE1 expression is upregulated in AML. PRICKLE1 is known to be involved in PCP, including convergent extension and cell migration [32]. The overexpression of PRICKLE1 has been found to be associated with poor survival in several solid tumors. In accordance with our findings, previous studies have revealed that the mRNA level of PRICKLE1 was substantially elevated in solid tumors, such as basal breast cancers [3], and triple-negative breast cancers [12]. More importantly, our data revealed that PRICKLE1 expression decreased after complete remission and reincreased during relapse phase. Of note, the expression of PRICKLE1 is significantly higher in resistant AML cell lines than sensitive AML cell lines, suggesting that PCP proteins PRICKLE1 may be involved in drug resistance through regulating cell polarity and movements. In addition, the expression level of PRICKLE1 in monocytic-AML was higher than that in granulocytic-AML. We noticed that PRICKLE1 mRNA and protein levels were much higher in AML patients with extramedullary metastasis (Fig. 3d), especially in patients with central nervous system leukemia (CNSL). These results suggested that PRICKLE1 expression could be associated with therapeutic efficacy.

Moreover, we observed that high PRICKLE1 expression was associated with higher BM blasts, more unfavorable gene mutation and poorer ELN risk classification in AML. Survival analysis revealed that patients with high PRICKLE1 expression had a poor prognosis in whole cohort AML and non-M3 AML. In addition, our study analyzed prognostic significance of PRICKLE1 in TCGA-LAML data (represents adult AML) and TARGET-AML data (represents children and adolescents AML). The results showed that PRICKLE1^high^ cases had shorter OS (TCGA-LAML: P = 0.055; TARGET-AML: P < 0.001) than PRICKLE1^low^ cases. It seems that PRICKLE1 has a better indication of prognostic significance in children and adolescents AML than in adult AML. More importantly, we can reach the same conclusion that high PRICKLE1 expression is an independent prognostic indicator of adverse outcomes in adults and children with AML. We also found that PRICKLE1 was an independent prognostic factor for OS based on univariate and multivariate analyses. Besides, age and cytogenetic risk classification were prognostic factors based on univariate analysis. Consequently, it is considered that PRICKLE1 plays an important role in disease progression. Hence, PRICKLE1 expression could be used to predict inferior survival and assess treatment outcome in AML.

PCP signalling is crucial for tissue morphogenesis and depends on a group of core proteins Frizzled (FZD), VANGL, Disheveled (DVL) and PRICKLE [10]. Human WNT5A, WNT5B, and WNT11 are representative non-canonical WNTs transducing PCP signals through FZD3 or FZD6 receptors, and ROR1, ROR2 or PTK7 co-receptors. Human VANGLs, CELSRs, DVLs, PRICKLE1 and PRICKLE2 are core PCP signaling molecules [9, 24]. Our results indicated that the core Wnt/PCP pathway components DVL2, PRICKLE1, LEF1 and active β-catenin were upregulated in AML patients. To explore the functional roles of PRICKLE1, we analyzed PRICKLE1 centered gene network and protein–protein interaction network using GeneMANIA and STRING online tools, respectively. Some of the interaction between molecules have been verified experimentally, such as SMURF2 [33], VANGL1, VANGL2 [34] and DVL2 [35, 36]. However, we are very interesting to study about the interaction remains unknown in future, such as PRICKLE1 interacted with PARD6A.

Our study analyzed the DEGs in four AML patients and three normal controls. FLT3 gene was one of the high expression group in the DEGs of our study, which is accordance with previous studies [37–41]. Besides, the expression of FLT3 in AML was indeed increased, however, there was no significant difference in the expression of FLT3 between the PRICKLE1^high^ and PRICKLE1^low^ groups by using the TCGA-LAML database (Additional file 1: Fig. S4). We identified that high PRICKLE1 expression represents poor survival in AML patients, and is associated with FLT3-ITD mutation and other known mutations. We explored PRICKLE1 expression and its prognostic significance, in particular focuses on the relationship between PRICKLE1 and Wnt signalling and metastasis/invasion. Collectively, our analysis data here strongly suggest the role of PRICKLE1 in the Wnt/PCP pathway of AML. This study showed PRICKLE1 was significantly upregulated in AML patients, which suggested that inhibition of PRICKLE1 is a potential therapeutic strategy in AML.

## Conclusions

In conclusion, we comprehensively analyzed the expression of PRICKLE1 in AML patients and cell lines using our data and data online. Our results indicated that PRICKLE1 is overexpressed in AML patients, and its high expression is correlated with adverse risk factors. Moreover, high expression of PRICKLE1 was found in FLT3/DNMT3A/IDH1/IDH2-mutant AML and associated with poor prognosis. More importantly, PRICKLE1 may mediate migration and invasion through the Wnt/PCP signaling pathway. Overall, here we show that the key PCP pathway component PRICKEL1 is upregulated in AML cells. We show that patients with high expression of PRICKLE1, have a less favorable clinical prognosis. Our findings would help to better understand the role of PRICKLE1 in chemoresistance and progression of AML and highlight the unique function of PRICKLE1 as a candidate gene for prognostic biomarker and therapeutic target.

## Supplementary Information

**Additional file 1.**. Figure S1. qRT-PCR and western blotting analysis of PRICKLE1 expression in 18 AML patients; Figure S2. Association of PRICKLE1 expression with FAB classifications monocytic-AML (AML-M5) and granulocytic-AML (AML-M1/M2/M3), and NPM1 gene mutation; Figure S3. Overall survival (OS) of CN-AML patients according to PRICKEL1 expression in our study and in TCGA-LAML data; Figure S4. Comparision of the FLT3 expression levels between the PRICKLE1^high^ and PRICKLE1^low^ groups by using the TCGA-LAML database; Table S1. Fourteen differentially expressed genes between AML patients and healthy controls; Table S2. Patients’ information in western blotting; Table S3. Sixteen differentially expressed genes between AML patients and healthy controls.

## Data Availability

The data that support the findings of this study are available from the corresponding author upon reasonable request.

## Abbreviations

AML: Acute myeloid leukemia
PCP: Planar cell polarity
PRICKLE1: Prickle planar cell polarity protein 1
RNA-seq: RNA-sequencing
GEPIA: Gene Expression Profiling Interactive Analysis
TCGA: The Cancer Genome Atlas
TARGET: Therapeutically Applicable Research to Generate Effective Treatments
STRING: Search Tool for the Retrieval of Interacting Genes
FAB: French-American-British
WHO: World Health Organization
CR: Complete remission
ELN: European LeukemiaNet
BM: Bone marrow
qRT-PCR: Quantitative reverse transcription-polymerase chain reaction
EFS: Event-free survival
OS: Overall survival
DEGs: Differentially expressed genes
CN-AML: Cytogenetically normal AML
EMT: Epithelial-to-mesenchymal transition
LSCs: Leukemia stem cells
CNSL: Central nervous system leukemia
