# Supplementary material for: PRICKLE1, a Wnt/PCP signaling component, is overexpressed and associated with inferior prognosis in acute myeloid leukemia
Source: J Transl Med. 2021 May 17;19:211. doi: 10.1186/s12967-021-02873-8 (PMC8130533; doi:10.1186/s12967-021-02873-8)
Supplement: Supplementary file 1 — Additional file 1.. Figure S1. qRT-PCR and western blotting analysis of PRICKLE1 expression in 18 AML patients; Figure S2. Association of PRICKLE1 expression with FAB classifications monocytic-AML (AML-M5) and granulocytic-AML (AML-M1/M2/M3), and NPM1 gene mutation; Figure S3. Overall survival (OS) of CN-AML patients according to PRICKEL1 expression in our study and in TCGA-LAML data; Figure S4. Comparision of the FLT3 expression levels between the PRICKLE1high and PRICKLE1low groups by using the TCGA-LAML database; Table S1. Fourteen differentially expressed genes between AML patients and healthy controls; Table S2. Patients’ information in western blotting; Table S3. Sixteen differentially expressed genes between AML patients and healthy controls. [file 12967_2021_2873_MOESM1_ESM.doc]

PRICKLE1, a Wnt/PCP signaling component, is overexpressed and associated with inferior prognosis in acute myeloid leukemia

Duanfeng Jiang^1^, Yanjuan He^2^, Qiuyu Mo^3^, Enyi Liu^2^, Xin Li^1^, Lihua Huang^4^, Qin Zhang^1^, Fangping Chen^1,2^, Yan Li^2^**^*^**, Haigang Shao^1^**^*^**

^1^ Department of Hematology, The 3rd Xiangya Hospital, Central South University, Changsha, Hunan, China; ^2^ Department of Hematology, Xiangya Hospital, Central South University, Changsha, China; ^3^ Department of Hematology, Guilin People's Hospital, Guilin Medical College, Guilin, China; ^4^ Center for Medical Experiments, The 3rd Xiangya Hospital of Central South University, Changsha, China.

**Supplementary figures**

**
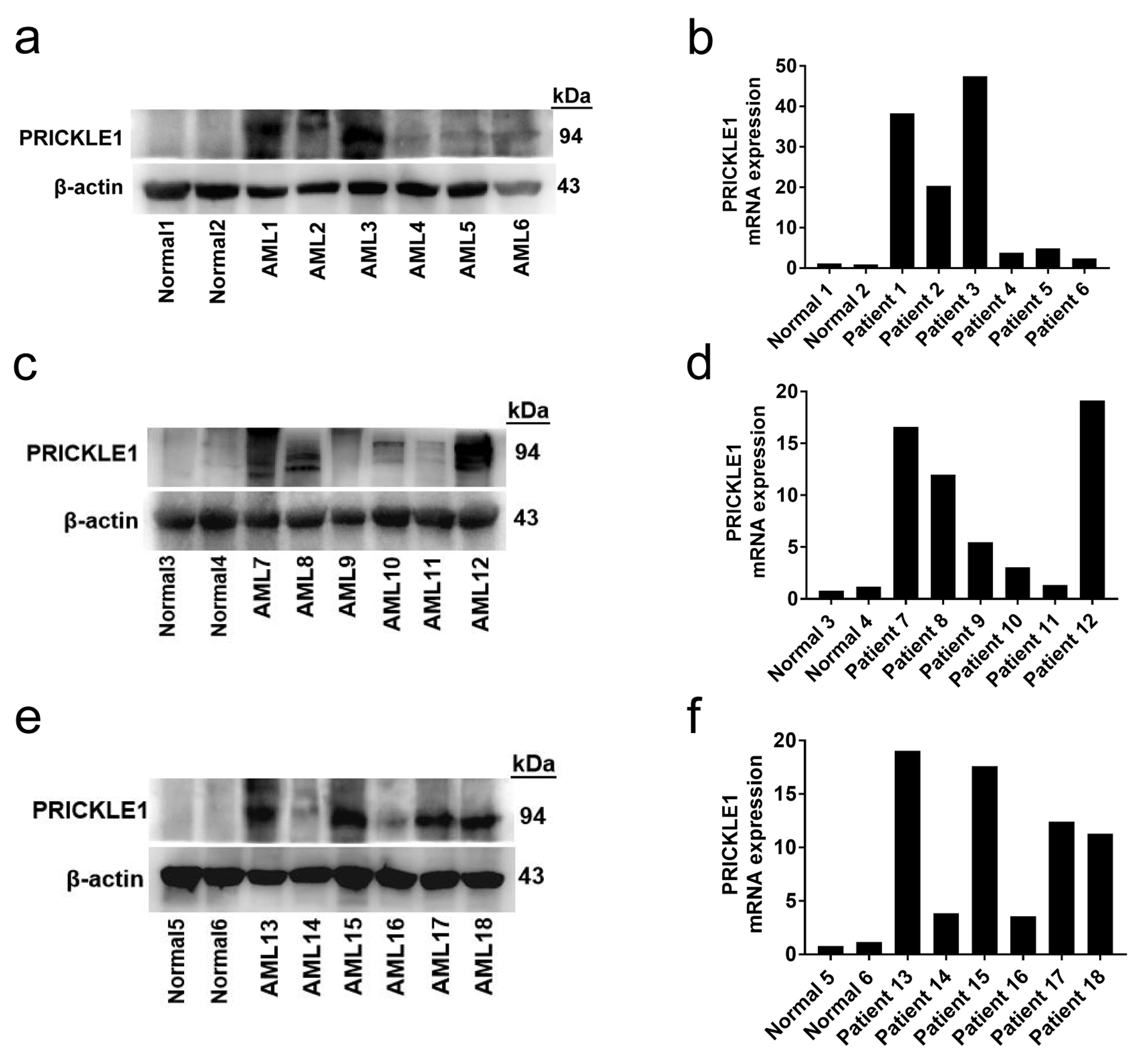
**

**Figure S1.** (**a-f**) qRT-PCR and western blotting analysis of PRICKLE1 expression in 18 AML patients.

**
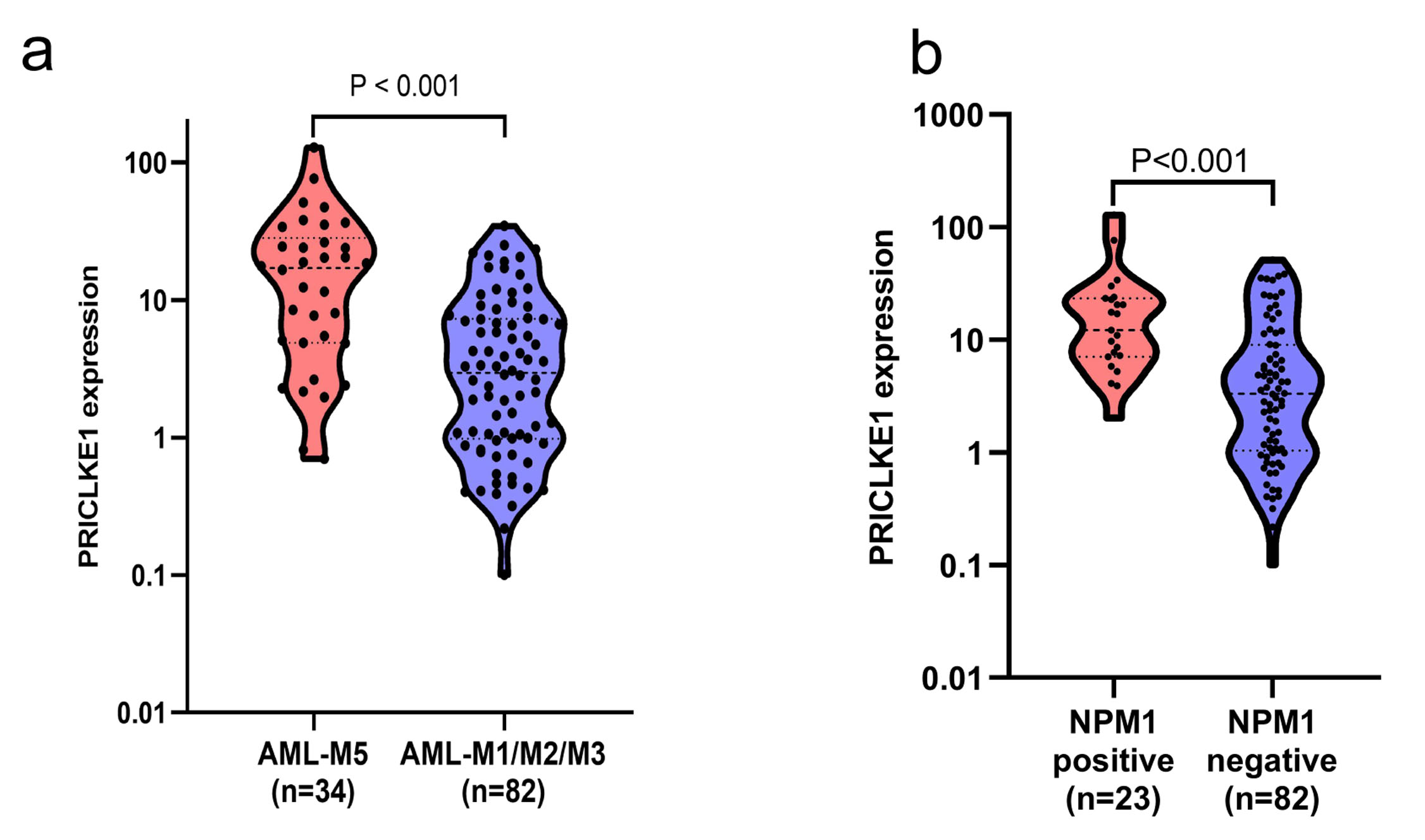
**

**Figure S2.** (**a**) Association of PRICKLE1 expression with French-American-British (FAB) classifications monocytic-AML (AML-M5) and granulocytic-AML (AML-M1/M2/M3). (**b**) Association of PRICKLE1 expression with NPM1 gene mutation.

**
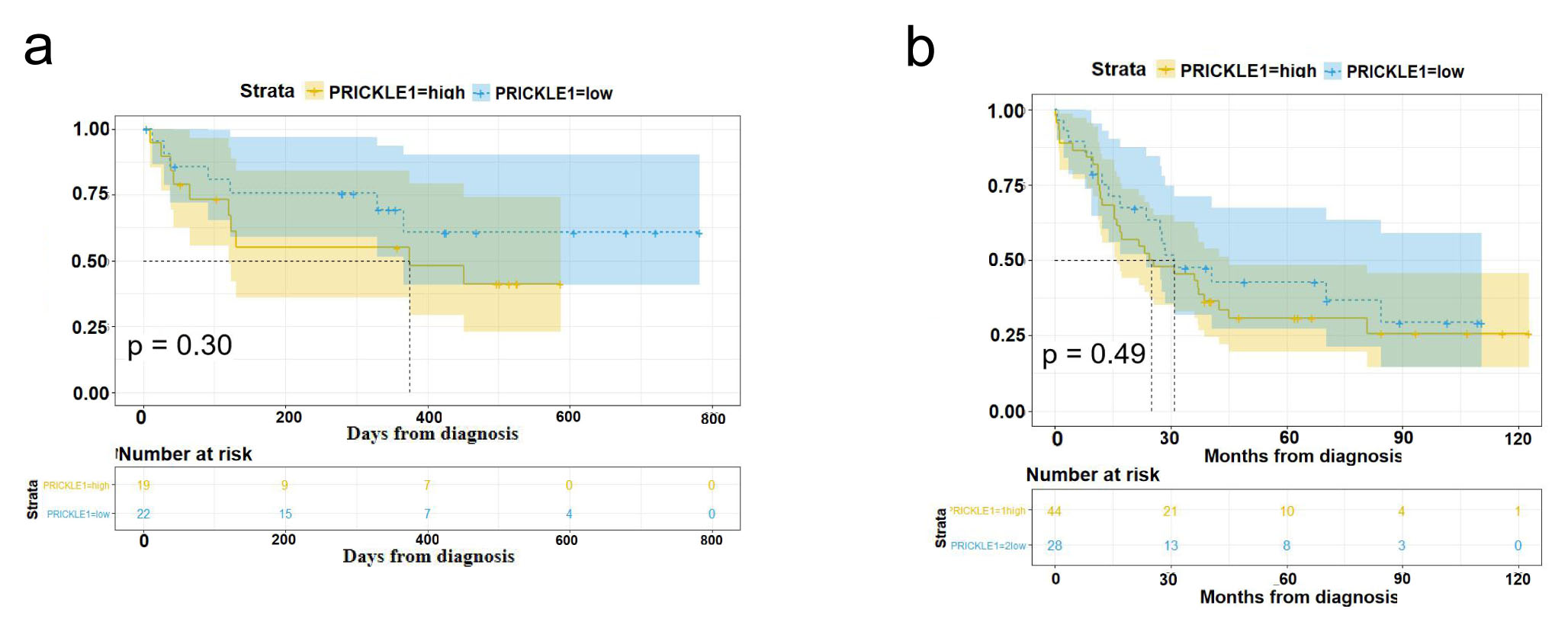
**

**Figure S3.** Survival analysis of acute myeloid leukemia (AML) patients according to PRICKEL1 expression. (**a**) Overall survival (OS) of cytogenetically normal AML (CN-AML) patients in our study. (**b**) OS of CN-AML patients in TCGA-LAML data.

**Figure S4.** There was no significant difference in the expression of FLT3 between the PRICKLE1^high^ and PRICKLE1^low^ groups by using the TCGA-LAML database.

**Supplementary tables**

Table S1. Fourteen differentially expressed genes between AML patients and healthy controls.

| **Gene ID** | **Gene Symbol** | **log2FC** | **Qvalue** | **Up/down** |
| --- | --- | --- | --- | --- |
| ENSG00000137393 | RNF144B | -2.641784086 | 1.88E-04 | Down |
| ENSG00000138639 | ARHGAP24 | -2.500625843 | 6.43E-05 | Down |
| ENSG00000163162 | RNF149 | -2.11063445 | 1.09E-02 | Down |
| ENSG00000100852 | ARHGAP5 | -2.304908659 | 4.98E-03 | Down |
| ENSG00000168646 | AXIN2 | -3.882599036 | 1.65E-04 | Down |
| ENSG00000188064 | WNT7B | 4.521117456 | 1.41E-02 | Up |
| ENSG00000180340 | FZD2 | 2.113179614 | 1.08E-03 | Up |
| ENSG00000139174 | PRICKLE1 | 4.959923773 | 2.62E-17 | Up |
| ENSG00000075275 | CELSR1 | 3.287842159 | 5.31E-08 | Up |
| ENSG00000076716 | GPC4 | 2.346278557 | 1.32E-02 | Up |
| ENSG00000147257 | GPC3 | 7.089982664 | 1.83E-04 | Up |
| ENSG00000184937 | WT1 | 7.418318236 | 1.75E-05 | Up |
| ENSG00000122025 | FLT3 | 5.061451335 | 1.91E-25 | Up |
| ENSG00000115596 | WNT6 | 6.036086079 | 2.01E-03 | Up |

1. Table S2. Patients’ information in western blotting.

| **Patient** | **Sex** | **Age** | **FAB subtype** | **BM blasts** | **Karyotypes** | **Cytogenetic risk** | **ELN risk stratification** |
| --- | --- | --- | --- | --- | --- | --- | --- |
| Patient 1 | Female | 36 | M5b | 87 | t(9;11) | Intermediate | Intermediate |
| Patient 2 | Male | 39 | M2a | 93 | 11q23 | Poor | Poor |
| Patient 3 | Male | 59 | M5a | 75 | Complex | Poor | Poor |
| Patient 4 | male | 76 | M2b | 83 | Normal | Intermediate | Favorable |
| Patient 5 | female | 34 | M4b | 82 | Normal | Intermediate | Favorable |
| Patient 6 | female | 54 | M4Eo | 75 | inv(16) | Favorable | Favorable |
| Patient 7 | female | 65 | M2a | 91 | Normal | Intermediate | Intermediate |
| Patient 8 | male | 77 | M5b | 93 | 11q23 | Poor | Poor |
| Patient 9 | male | 53 | M2a | 88 | t(8;21) | Favorable | Favorable |
| Patient 10 | male | 54 | M2a | 94 | Normal | Intermediate | Favorable |
| Patient 11 | female | 48 | M2a | 82 | t(8;21) | Favorable | Intermediate |
| Patient 12 | female | 51 | M5b | 92 | Normal | Intermediate | Intermediate |
| Patient 13 | female | 54 | M5a | 72 | Other | Poor | Poor |
| Patient 14 | female | 13 | M2b | 62 | t(8;21) | Favorable | Favorable |
| Patient 15 | male | 27 | M5b | 90 | 11q23 | Poor | Poor |
| Patient 16 | female | 61 | M1 | 80 | Normal | Intermediate | Favorable |
| Patient 17 | female | 69 | M5b | 93 | Normal | Intermediate | Intermediate |
| Patient 18 | female | 60 | M5b | 60 | Normal | Intermediate | Poor |

Note: Patient 1, 2 and 3 with extramedullary disease myeloid sarcoma (MS), MS and leukemia cutis (LC), respectively.

Table S3. Sixteen differentially expressed genes between AML patients and healthy controls.

| **Gene ID** | **Gene Symbol** | **log2FC** | **Qvalue** | **Up/down** |
| --- | --- | --- | --- | --- |
| ENSG00000039068 | CDH1 | -2.458377909 | 2.23E-02 | Down |
| ENSG00000168421 | RHOH | -2.896805698 | 1.58E-03 | Down |
| ENSG00000138639 | ARHGAP24 | -2.500625843 | 6.43E-05 | Down |
| ENSG00000137393 | RNF144B | -2.641784086 | 1.88E-04 | Down |
| ENSG00000163162 | RNF149 | -2.11063445 | 1.09E-02 | Down |
| ENSG00000100852 | ARHGAP5 | -2.304908659 | 4.98E-03 | Down |
| ENSG00000168646 | AXIN2 | -3.882599036 | 1.65E-04 | Down |
| ENSG00000106178 | CCL24 | 4.355957901 | 4.74E-02 | Up |
| ENSG00000137673 | MMP7 | 4.629049056 | 3.78E-02 | Up |
| ENSG00000157227 | MMP14 | 3.352501374 | 3.43E-10 | Up |
| ENSG00000087245 | MMP2 | 3.01709369 | 1.78E-05 | Up |
| ENSG00000102962 | CCL22 | 4.722771454 | 1.08E-02 | Up |
| ENSG00000274736 | CCL23 | 3.052635596 | 2.02E-02 | Up |
| ENSG00000139174 | PRICKLE1 | 4.959923773 | 2.62E-17 | Up |
| ENSG00000160678 | S100A1 | 6.009471416 | 3.26E-05 | Up |
| ENSG00000123342 | MMP19 | 2.155138997 | 9.16E-03 | Up |
